# Supplementary material for: Multi‐dataset identification of innovative feature genes and molecular mechanisms in keratoconus
Source: J Cell Mol Med. 2024 Sep 19;28(18):e70079. doi: 10.1111/jcmm.70079 (PMC11412914; doi:10.1111/jcmm.70079)
Supplement: Supplementary file 2 — Material S2. [file JCMM-28-e70079-s002.zip › 7.GSEA analysis/6.SNORA74B/KEGG_AXON_GUIDANCE.html]

Details for gene set KEGG\_AXON\_GUIDANCE[GSEA]

|  || Dataset | SNORA74B.group.cls#h\_versus\_l |
| Phenotype | group.cls#h\_versus\_l |
| Upregulated in class | l |
| GeneSet | KEGG\_AXON\_GUIDANCE |
| Enrichment Score (ES) | -0.35400233 |
| Normalized Enrichment Score (NES) | -1.3517677 |
| Nominal p-value | 0.0831758 |
| FDR q-value | 0.31768408 |
| FWER p-Value | 0.93 |
Table: GSEA Results Summary

  

Fig 1: Enrichment plot: KEGG\_AXON\_GUIDANCE      
 Profile of the Running ES Score & Positions of GeneSet Members on the Rank Ordered List

  

| SYMBOL | TITLE | RANK IN GENE LIST | RANK METRIC SCORE | RUNNING ES | CORE ENRICHMENT || 1 | EPHA8 | na | 356 | 0.663 | 0.0209 | No |
| 2 | UNC5A | na | 960 | 0.494 | 0.0267 | No |
| 3 | NFATC2 | na | 1406 | 0.425 | 0.0338 | No |
| 4 | RAC3 | na | 1795 | 0.390 | 0.0409 | No |
| 5 | PPP3R2 | na | 2054 | 0.366 | 0.0506 | No |
| 6 | SEMA6C | na | 2767 | 0.316 | 0.0449 | No |
| 7 | NTN3 | na | 3669 | 0.272 | 0.0316 | No |
| 8 | CHP2 | na | 4857 | 0.230 | 0.0081 | No |
| 9 | ABLIM2 | na | 4934 | 0.227 | 0.0166 | No |
| 10 | ABLIM3 | na | 5114 | 0.222 | 0.0218 | No |
| 11 | EPHA6 | na | 5149 | 0.221 | 0.0312 | No |
| 12 | ROBO2 | na | 5828 | 0.198 | 0.0209 | No |
| 13 | EPHA4 | na | 5866 | 0.197 | 0.0291 | No |
| 14 | SEMA5B | na | 6601 | 0.173 | 0.0160 | No |
| 15 | EPHB1 | na | 7283 | 0.154 | 0.0036 | No |
| 16 | ABLIM1 | na | 7677 | 0.144 | -0.0010 | No |
| 17 | GNAI3 | na | 7691 | 0.144 | 0.0054 | No |
| 18 | SEMA3E | na | 7720 | 0.143 | 0.0113 | No |
| 19 | RHOD | na | 8082 | 0.134 | 0.0071 | No |
| 20 | NCK2 | na | 8122 | 0.133 | 0.0122 | No |
| 21 | RGS3 | na | 8402 | 0.127 | 0.0101 | No |
| 22 | ROCK1 | na | 8851 | 0.117 | 0.0027 | No |
| 23 | PLXNA1 | na | 9174 | 0.110 | -0.0015 | No |
| 24 | RASA1 | na | 9229 | 0.109 | 0.0021 | No |
| 25 | UNC5C | na | 9345 | 0.106 | 0.0037 | No |
| 26 | SEMA3F | na | 9636 | 0.100 | 0.0000 | No |
| 27 | KRAS | na | 9766 | 0.097 | 0.0009 | No |
| 28 | NTN1 | na | 10165 | 0.089 | -0.0064 | No |
| 29 | LIMK1 | na | 10417 | 0.085 | -0.0097 | No |
| 30 | SEMA4C | na | 10658 | 0.080 | -0.0128 | No |
| 31 | PLXNA2 | na | 11400 | 0.068 | -0.0310 | No |
| 32 | EPHA1 | na | 11568 | 0.066 | -0.0328 | No |
| 33 | SEMA4D | na | 12198 | 0.057 | -0.0483 | No |
| 34 | SEMA3B | na | 12210 | 0.057 | -0.0459 | No |
| 35 | PLXNB3 | na | 12373 | 0.055 | -0.0480 | No |
| 36 | SEMA4G | na | 12804 | 0.049 | -0.0581 | No |
| 37 | SRGAP3 | na | 13276 | 0.044 | -0.0697 | No |
| 38 | EPHB3 | na | 13637 | 0.040 | -0.0782 | No |
| 39 | GNAI2 | na | 14445 | 0.031 | -0.1000 | No |
| 40 | UNC5B | na | 14498 | 0.030 | -0.1001 | No |
| 41 | LRRC4C | na | 14523 | 0.030 | -0.0994 | No |
| 42 | EFNA1 | na | 14655 | 0.028 | -0.1019 | No |
| 43 | ROBO1 | na | 15690 | 0.018 | -0.1309 | No |
| 44 | EPHA5 | na | 16135 | 0.013 | -0.1431 | No |
| 45 | EPHB4 | na | 16171 | 0.013 | -0.1435 | No |
| 46 | MAPK3 | na | 16753 | 0.007 | -0.1600 | No |
| 47 | SEMA4A | na | 17147 | 0.003 | -0.1712 | No |
| 48 | PAK4 | na | 17175 | 0.003 | -0.1719 | No |
| 49 | PLXNB1 | na | 17645 | -0.002 | -0.1853 | No |
| 50 | ROCK2 | na | 17655 | -0.002 | -0.1855 | No |
| 51 | CFL1 | na | 17798 | -0.004 | -0.1894 | No |
| 52 | GSK3B | na | 17871 | -0.004 | -0.1913 | No |
| 53 | SEMA6D | na | 18102 | -0.007 | -0.1976 | No |
| 54 | PLXNB2 | na | 18192 | -0.008 | -0.1998 | No |
| 55 | RHOA | na | 18290 | -0.009 | -0.2021 | No |
| 56 | CDC42 | na | 18770 | -0.015 | -0.2153 | No |
| 57 | RAC2 | na | 18892 | -0.016 | -0.2180 | No |
| 58 | PAK6 | na | 18984 | -0.017 | -0.2199 | No |
| 59 | DCC | na | 19371 | -0.022 | -0.2300 | No |
| 60 | EFNB1 | na | 19482 | -0.023 | -0.2321 | No |
| 61 | PPP3R1 | na | 19504 | -0.023 | -0.2317 | No |
| 62 | SEMA6B | na | 19593 | -0.024 | -0.2331 | No |
| 63 | HRAS | na | 19699 | -0.025 | -0.2349 | No |
| 64 | RAC1 | na | 19881 | -0.027 | -0.2389 | No |
| 65 | EFNB2 | na | 19919 | -0.028 | -0.2387 | No |
| 66 | NFATC3 | na | 20022 | -0.029 | -0.2403 | No |
| 67 | EFNA2 | na | 20126 | -0.030 | -0.2418 | No |
| 68 | MAPK1 | na | 20606 | -0.035 | -0.2540 | No |
| 69 | SRGAP1 | na | 20915 | -0.039 | -0.2611 | No |
| 70 | DPYSL5 | na | 20942 | -0.039 | -0.2600 | No |
| 71 | PAK3 | na | 21207 | -0.042 | -0.2657 | No |
| 72 | CHP1 | na | 21507 | -0.046 | -0.2721 | No |
| 73 | CDK5 | na | 21681 | -0.048 | -0.2749 | No |
| 74 | PPP3CB | na | 21942 | -0.051 | -0.2800 | No |
| 75 | SEMA4B | na | 22138 | -0.053 | -0.2831 | No |
| 76 | PLXNA3 | na | 22366 | -0.056 | -0.2870 | No |
| 77 | EFNA4 | na | 22531 | -0.059 | -0.2890 | No |
| 78 | EFNA5 | na | 22595 | -0.060 | -0.2880 | No |
| 79 | PTK2 | na | 22693 | -0.061 | -0.2880 | No |
| 80 | PPP3CA | na | 23024 | -0.066 | -0.2944 | No |
| 81 | NCK1 | na | 23215 | -0.069 | -0.2967 | No |
| 82 | ABL1 | na | 23665 | -0.076 | -0.3061 | No |
| 83 | CXCL12 | na | 23758 | -0.077 | -0.3052 | No |
| 84 | SLIT1 | na | 23993 | -0.081 | -0.3081 | No |
| 85 | LIMK2 | na | 24245 | -0.085 | -0.3114 | No |
| 86 | ROBO3 | na | 24687 | -0.093 | -0.3198 | No |
| 87 | NFAT5 | na | 24769 | -0.094 | -0.3177 | No |
| 88 | SRGAP2 | na | 25613 | -0.109 | -0.3369 | No |
| 89 | PAK2 | na | 25754 | -0.112 | -0.3357 | No |
| 90 | L1CAM | na | 26099 | -0.119 | -0.3401 | No |
| 91 | ARHGEF12 | na | 26140 | -0.120 | -0.3356 | No |
| 92 | EPHA2 | na | 26144 | -0.120 | -0.3301 | No |
| 93 | SLIT3 | na | 26312 | -0.123 | -0.3291 | No |
| 94 | SEMA5A | na | 26780 | -0.134 | -0.3363 | No |
| 95 | EPHA7 | na | 27253 | -0.145 | -0.3431 | No |
| 96 | ITGB1 | na | 27543 | -0.152 | -0.3443 | No |
| 97 | NFATC4 | na | 27880 | -0.162 | -0.3464 | Yes |
| 98 | NRAS | na | 27969 | -0.164 | -0.3413 | Yes |
| 99 | EPHB6 | na | 28369 | -0.176 | -0.3445 | Yes |
| 100 | GNAI1 | na | 28399 | -0.176 | -0.3371 | Yes |
| 101 | PAK1 | na | 28478 | -0.179 | -0.3309 | Yes |
| 102 | PPP3CC | na | 28713 | -0.186 | -0.3290 | Yes |
| 103 | SEMA3A | na | 28731 | -0.187 | -0.3207 | Yes |
| 104 | MET | na | 28809 | -0.189 | -0.3140 | Yes |
| 105 | NGEF | na | 29073 | -0.197 | -0.3124 | Yes |
| 106 | SEMA3G | na | 29141 | -0.199 | -0.3049 | Yes |
| 107 | EPHB2 | na | 29253 | -0.202 | -0.2986 | Yes |
| 108 | EPHA3 | na | 29303 | -0.204 | -0.2905 | Yes |
| 109 | EFNA3 | na | 30655 | -0.251 | -0.3177 | Yes |
| 110 | SEMA6A | na | 31250 | -0.281 | -0.3217 | Yes |
| 111 | EFNB3 | na | 31955 | -0.321 | -0.3270 | Yes |
| 112 | UNC5D | na | 32439 | -0.350 | -0.3245 | Yes |
| 113 | NTNG1 | na | 32446 | -0.351 | -0.3082 | Yes |
| 114 | FES | na | 32716 | -0.370 | -0.2986 | Yes |
| 115 | NRP1 | na | 33058 | -0.400 | -0.2897 | Yes |
| 116 | SEMA7A | na | 33233 | -0.417 | -0.2751 | Yes |
| 117 | NFATC1 | na | 33261 | -0.420 | -0.2561 | Yes |
| 118 | SEMA3D | na | 33277 | -0.422 | -0.2367 | Yes |
| 119 | CFL2 | na | 33537 | -0.454 | -0.2229 | Yes |
| 120 | FYN | na | 33540 | -0.454 | -0.2016 | Yes |
| 121 | PLXNC1 | na | 33619 | -0.467 | -0.1819 | Yes |
| 122 | CXCR4 | na | 33720 | -0.481 | -0.1622 | Yes |
| 123 | SLIT2 | na | 33899 | -0.512 | -0.1433 | Yes |
| 124 | SEMA4F | na | 34221 | -0.587 | -0.1250 | Yes |
| 125 | RND1 | na | 34308 | -0.616 | -0.0985 | Yes |
| 126 | NTN4 | na | 34361 | -0.637 | -0.0701 | Yes |
| 127 | DPYSL2 | na | 34371 | -0.640 | -0.0403 | Yes |
| 128 | SEMA3C | na | 34710 | -1.077 | 0.0005 | Yes |
Table: GSEA details [plain text format]

  

Fig 2: KEGG\_AXON\_GUIDANCE      
 Blue-Pink O' Gram in the Space of the Analyzed GeneSet

  

Fig 3: KEGG\_AXON\_GUIDANCE: Random ES distribution      
 Gene set null distribution of ES for **KEGG\_AXON\_GUIDANCE**
